# Prevalence and antimicrobial resistance of *Shigella flexneri* serotype 2 variant in China

**DOI:** 10.3389/fmicb.2015.00435

**Published:** 2015-05-07

**Authors:** Xianyan Cui, Jian Wang, Chaojie Yang, Beibei Liang, Qiuxia Ma, Shengjie Yi, Hao Li, Hongbo Liu, Peng Li, Zhihao Wu, Jing Xie, Leili Jia, Rongzhang Hao, Ligui Wang, Yuejin Hua, Shaofu Qiu, Hongbin Song

**Affiliations:** ^1^Nuclear-Agricultural Sciences, Zhejiang UniversityHangzhou, China; ^2^Institute of Disease Control and Prevention, Academy of Military Medical SciencesBeijing, China

**Keywords:** *Shigella flexneri*, serotype 2 variant, multidrug-resistant, antibiotic resistance rate, epidemic frequency

## Abstract

*Shigella flexneri* serotype 2 variant (II:3,4,7,8) was isolated in 2008 and first reported in China in 2013. In the present study, epidemiological surveillance from 2003 to 2013 in China suggested that this serotype first appeared in Guangxi in 2003; it then emerged in Shanghai and Xinjiang in 2004 and in Henan in 2008. Of the 1813 *S*. *flexneri* isolates, 58 *S. flexneri* serotype 2 variant strains were identified. Serotype 2 variant has emerged as a prominent serotype in recent years, with 2a (32.6%), X variant (25.2%), 1a (9.4%), X (6.3%), 2b (5.4%), and 1b (3.6%). According to phenotypic and genotypic analysis, the serotype 2 variant originated from 2a to 2b. A higher antibiotic resistance rate was observed between 2009 and 2013 than that between 2003 and 2008. Among 22 cephalosporin-resistant isolates, *bla*_TEM-1_, *bla*_OXA-1_, *bla*_CTX-3_, *bla*_CTX-14_, and *bla*_CTX-79_ were detected. Among 22 fluoroquinolone-resistant isolates, a Ser80Ile mutation in parC was present in all of the isolates. Moreover, 21 isolates had three gyrA point mutations (Ser83Leu, His211Tyr, Asp87Asn, or Gly) and one isolate had two gyrA point mutations (Ser83Leu and His211Tyr). The prevalence of His211Tyr in the fluoroquinolone-resistant isolates is concerning, and the mutation was first reported in China. Besides, 22 isolates harbored the *aac(6′)-Ib-cr* gene, and two isolates harbored *qnrS1*. In view of the increased epidemic frequency and multidrug-resistant strain emergence, continuous surveillance will be needed to understand the actual disease burden and provide guidance for shigellosis.

## Introduction

Shigellosis, caused by *Shigella* species, is recognized as a major public health burden and continues to be an important cause of diarrheal diseases. Worldwide, *Shigella* episodes were estimated to be 164.7 million annually, of which, 163.2 million were in developing countries, resulting in 1.1 million deaths (Kotloff et al., 1999). Epidemics generally occur in underdeveloped and developing countries with poor sanitary conditions where transmission from person to person is common, or when food or water is contaminated by the organism (Qu et al., 2012). Despite economic and public health improvements, outbreaks of shigellosis are still reported regularly (Huang et al., 2005; Gaynor et al., 2009; Kuo et al., 2009; Qiu et al., 2011). In China, shigellosis has been ranked third in morbidity, and it has become the number one cause of disease-related death in children (Mathers et al., 2009).

Based on biochemical and serological properties, the genus *Shigella* is divided into four species or subgroups: *S. flexneri*, *S. dysenteriae*, *S. boydii*, and *S. sonnei*. Among these subgroups, the epidemic subgroup *S. sonnei* caused diarrhea in industrialized countries and *S. flexneri* in developing countries (Kotloff et al., 1999). A previous study showed that the annual shigellosis morbidity rate was 20.28 cases per 100,000 people in mainland China from the national surveillance data of 2009, and *S. flexneri* (67.3%) and *S. sonnei* (32.7%) were two major causative species (Sui et al., 2010). *S. flexneri* is divided into at least 20 serotypes (serotypes 1a, 1b, 1c, 1d, 2a, 2b, 2 variant, 3a, 3b, 4a, 4av, 4b, 5a, 5b, X, Xv, Y, Yv, F6, and 7b) based on the combinations of antigenic determinants present on the O antigen of the cell envelope lipopolysaccharide (Qiu et al., 2013; Sun et al., 2013). Serotypes 1c, 4av, 7b, 1d, Yv, 2 variant and Xv are newly reported serotypes in recent years, and some have caused epidemic-level disease (Talukder et al., 2001; Stagg et al., 2008; Ye et al., 2010). Serotype 1c, first appeared in Bangladesh in 1989, was subsequently found to be prevalent in Bangladesh, Egypt, and Vietnam (Carlin et al., 1989; El-Gendy et al., 1999; Stagg et al., 2008). Serotype Xv was first identified in 2000 and later become the predominant serotype in Henan Province between 2002 and 2006. Serotype Xv was also the most prevalent serotype in Gansu and Anhui in 2007 (Ye et al., 2010). Therefore, the rapid expansion and spread of novel serotypes pose a severe threat to public health in areas where shigellosis is endemic.

The *S. flexneri* serotype 2 variant (II:3,4,7,8) was first reported by Qiu et al. in 2013 (Qiu et al., 2013). During our routine surveillance of bacillary dysentery from 2003 to 2013, a total of 58 serotype 2 variant strains were identified. This novel serotype first appeared in Guangxi in 2003, and then emerged in other provinces. However, no study has yet extensively characterized the newly emerging strains of serotype 2 variant; therefore, an extensive study is needed to determine the emergence, antimicrobial resistance pattern, and epidemic trends of the serotype 2 variant.

## Materials and methods

### Bacterial isolates, serotyping, and biochemical characterization

Fresh stool samples from diarrhea patients with clinically suspected dysentery were collected in sentinel hospitals. Samples were cultured for *Shigella* by streaking directly onto *Salmonella–Shigella* agar and incubated at 37°C for 18 h. Resultant *Shigella* colonies (colorless, semitransparent, smooth, and moist circular colonies) were routinely grown in a 37°C incubator in Luria–Bertani agar plates (Qu et al., 2012). Then, the strains were submitted to our laboratory for further confirmation. This study was approved by the ethics committee of the Academy of Military Medical Sciences (China), and written approval was also obtained from the patients involved in this study. The isolates were confirmed using API 20E test strips (bioMerieux Vitek, Marcy-l'Etoile, France) following the manufacturer's recommendations. Serotypes of *Shigella* isolates were further determined with two serotyping kits: a commercially available kit (Denka Seiken, Tokyo, Japan) and monoclonal antibody reagents (Reagensia AB, Stockholm, Sweden).

### Multilocus sequence typing (MLST)

MLST of these isolates was carried out using the protocols described at http://www.shigatox.net/ecmlst/cgi-bin/index. The PCR amplification conditions were as follows: 94°C for 5 min; 33 cycles of 94°C for 30 s, 55°C for 30 s, and 72°C for 1 min; and 72°C for 10 min with *Ex Taq* DNA polymerase (Takara, Dalian, China). The PCR amplicons of 15 housekeeping genes were sequenced, and sequences were edited using SeqMan 7.0. Then, the sequences were uploaded to the EcMLST website for comparison, which allowed us to determine the sequence type.

### Antimicrobial susceptibility testing

The antimicrobial MICs of 21 antimicrobials including ceftazidime (CAZ), ceftriaxone (CRO), cefepime (FEP), cefoperazone (CFP), cefazolin (CFZ), cefoxitin (FOX), imipenem (IPM), nitrofurantoin (NIT), piperacillin (PIP), ampicillin (AMP), ticarcillin (TIC), tetracycline (TE), tobramycin (TO), gentamicin (GEN), amikacin (AK), aztreonam (ATM), chloramphenicol (C), ticarcillin/clavulanic acid (TIM), levofloxacin (LEV), norfloxacin (NOR), and trimethoprim/sulfamethoxazole (SXT) were determined by broth microdilution using a 96-well microtiter plate (Sensititre, Thermo Fisher Scientific Inc., West Sussex, United Kingdom) according to the manufacturer's instructions, and MIC values follow Clinical and Laboratory Standards Institute recommendations. An *Escherichia coli* (ATCC 25922) strain was used as the quality control strain.

### Pulsed-field gel electrophoresis (PFGE)

All isolates of *S. flexneri* were analyzed by PFGE according to the standard protocol for *S. flexneri* developed by the Centers for Disease Control and Prevention of the USA. *Salmonella enterica* serotype Braenderup H9812 was digested with *Xba*I and used as molecular weight standard. Slices of agarose plugs were digested with *Not*I (Takara, Dalian, China) at 37°C for 3 h. Electrophoresis was carried out in 1% agarose SeaKem Gold gel (Lonza, Rockland, ME, USE) with the CHEF Mapper system (Bio-Rad) with the following run parameters: 6 V/cm and a linear increase in switching times from 2.16 to 54.17 s over a period of 20 h. The interpretation of the PFGE patterns was performed with BioNumerics software version 6.0 (Applied Maths, Sint-Martens-Latem, Belgium). A tree indicating relative genetic similarity was constructed based on the unweighted pair group method of averages and a position tolerance of 1.2%.

### PCR amplification of the antibiotic-resistant determinants and integrons

To understand the underlying mechanism conferring resistance to third-generation cephalosporins, PCR assays (Ahmed et al., 2006; Pan et al., 2006; Matar et al., 2007; Galani et al., 2010; Tariq et al., 2012) were used to detect the presence of β-lactamase genes such as *bla*_SHV_, *bla*_TEM_, *bla*_OXA_, and *bla*_CTX−M_. The variable regions of class 1 integrons and class 2 integrons were also amplified with primers listed in Table 1. The primers hep58 and hep59, hep74 and hep51 were used to detect the variable regions of class 1 integrons and class 2 integrons, respectively. The reverse primers *aadA1*, *aadA2*, *aadA5*, and *cmlA1*, together with the forward primer hep58, were used to amplify the gene cassettes of class 1 integron-positive strains. The forward primer hep74 and reverse primer hep51 were used to amplify the gene cassettes of class 2 integron-positive strains. To understand the underlying mechanism conferring resistance to quinolones, plasmid-mediated quinolone resistance determinants (PMQRs) such as *qnrA*, *qnrB*, *qnrD*, *qnrS*, and *aac*(*6′)-Ib-cr* and quinolone resistance–determining regions (QRDRs) of the DNA gyrase (*gyrA*) and topoisomerase IV (*parC*) genes were amplified (Robicsek et al., 2006; Hu et al., 2007; Pu et al., 2009; Tariq et al., 2012). Purified PCR fragments were sequenced and analyzed by comparison with sequences in GenBank.

**Table 1:** **Primers for the antibiotic-resistant determinants and integrons**.

**Target**	**Primer sequence (5′–3′)**	**Amplicon size (bp)**	**References**
**β-Lactamases**			
*bla*CTX-M-1 group	F: GGTTAAAAAATCACTGCGTC	873	Matar et al., 2007
	R: TTACAAACCGTCGGTGACGA		
*bla*CTX-M-9 group	F: AGAGTGCAACGGATGATG	868	Matar et al., 2007
	R: CCAGTTACAGCCCTTCGG		
*bla*CTX-M-2/8/25 group	F: ACCGAGCCSACGCTCAA	221	This study
	R: CCGCTGCCGGTTTTATC		
*bla*TEM	F: ATGAGTATTCAACATTTCCG	1080	Tariq et al., 2012
	R: CCAATGCTTAATCAGTGAGG		
*bla*OXA	F: ATTAAGCCCTTTACCAAACCA	890	Ahmed et al., 2006
	R: AAGGGTTGGGCGATTTTGCCA		
*bla*VIM	F: AGTGGTGAGTATCCGACAG	509	Galani et al., 2010
	R: ATGAAAGTGCGTGGAGAC		
*bla*NDM	F: GTCTGGCAGCACACTTCCTA	515	This study
	R: TAGTGCTCAGTGTCGGCATC		
**INTEGRONS**			
IntI1	F: ACATGTGATGGCGACGCACGA	569	Pan et al., 2006
	R: ATTTCTGTCCTGGCTGGCGA		
Class 1 integron variable region	hep58: TCATGGCTTGTTATGACTGT	Variable	This study
	hep59:GTAGGGCTTATTATGCACGC		
Class 1 integron variable region	hep58: TCATGGCTTGTTATGACTGT	Variable	This study
	aadA1:TGTCAGCAAGATAGCCAGAT		
Class 1 integron variable region	hep58: TCATGGCTTGTTATGACTGT	Variable	This study
	aadA2: TGATCTCGCCTTTCACAAA		
Class 1 integron variable region	hep58: TCATGGCTTGTTATGACTGT	Variable	This study
	aadA5: CATCTAACGCATAGTTGAGC		
Class 1 integron variable region	hep58: TCATGGCTTGTTATGACTGT	Variable	This study
	cmlA1: CAACGATTGGGATTTGACGTACTTT		
IntI2	F: CACGGATATGCGACAAAAAGGT	789	Pan et al., 2006
	R: GTAGCAAACGAGTGACGAAATG		
Class 2 integron variable region	hep74: CGGGATCCCGGACGGCATGCACGATTTGTA	Variable	Ahmed et al., 2006
	hep51: GATGCCATCGCAAGTACGAG		
**QRDR OF TOPISOMERASE GENES**			
*gyrA*	F: TACACCGGTCAACATTGAGG	648	Hu et al., 2007
	R: TTAATGATTGCCGCCGTCGG		
*gyrB*	F: TGAAATGACCCGCCGTAAAGG	309	Hu et al., 2007
	R: GCTGTGATAACGCAGTTTGTCCGGG		
*parC*	F: GTACGTGATCATGGACCGTG	531	Hu et al., 2007
	R: TTCGGCTGGTCGATTAATGC		
*parE*	F: ATGCGTGCGGCTAAAAAAGTG	290	Hu et al., 2007
	R: TCGTCGCTGTCAGGATCGATAC		
**PMQR**			
*qnrA*	F: ATTTCTCACGCCAGGATTTG	516	Robicsek et al., 2006
	R: GATCGGCAAAGGTYAGGTCA		
*qnrB*	F: GATCGTGAAAGCCAGAAAGG	469	Robicsek et al., 2006
	R: ACGAYGCCTGGTAGTTGTCC		
*qnrD*	F: CGAGATCAATTTACGGGGAATA	656	Tariq et al., 2012
	R: AACAAGCTGAAGCGCCTG		
*qnrS*	F: ACGACATTCGTCAACTGCAA	417	Robicsek et al., 2006
	R: TAAATTGGCACCCTGTAGGC		
*aac(6′)-Ib-cr*	F: GCAACGCAAAAACAAAGTTAGG	560	Pu et al., 2009
	R: GTGTTTGAACCATGTACA		

## Results

### Bacteria isolation and biochemical characterization

**Figure 1:**
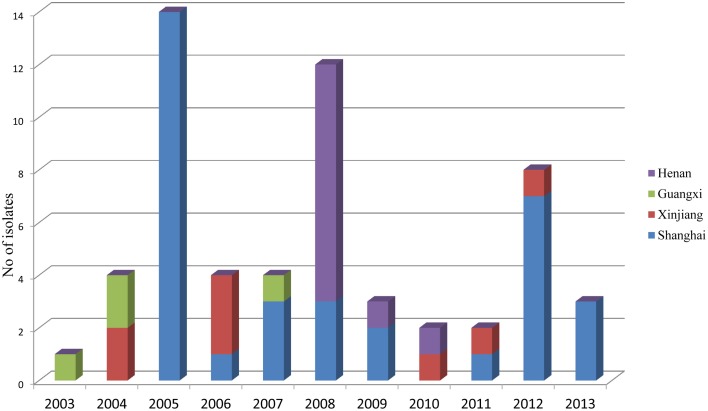
***Shigella flexneri* species distribution from 2003 to 2013 in China**.

**Figure 2:**
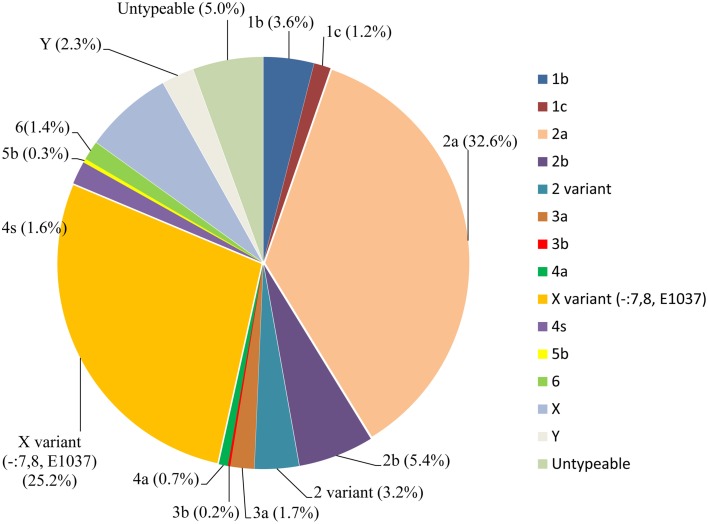
***S. flexneri* species proportions from 2003 to 2013 in China**.

During the routine surveillance of shigellosis in our laboratory, we screened 1813 *S. flexneri* isolates collected from the eastern, western, southern, northern and central regions of China during 2003–2013. In the 11-year surveillance of *Shigella* infections, a total of 58 *S. flexneri* serotype 2 variant isolates were identified. The serotype 2 variant first appeared in Guangxi Province in 2003, then emerged in Shanghai City and Xinjiang Province in 2004, and it was found in Henan Province in 2008. Among the 58 serotype 2 variant isolates, 34 isolates were collected from Shanghai City during the period 2005–2013, 12 isolates were collected from Henan Province during the period 2008–2010, eight isolates were collected from Xinjiang Province during the period 2004–2012, and four isolates were collected from Guangxi Province during the period 2003–2007 (Figure 1; see also Table S1 in the Supplemental Material). Notably, serotype 2 variant has emerged as a common serotype (3.2%), with 2a (32.6%), X variant (25.2%), 1a (9.4%), X (6.3%), 2b (5.4%) and 1b (3.6%) during 2003–2013 (Figure 2). In addition, all examined strains of *S. flexneri* serotype 2 variant displayed the same typical biochemical features of *Shigella* species with the ability to ferment glucose, arabinose, mannitol, and melibiose.

### Antimicrobial susceptibility testing

Fifty-eight *S. flexneri* serotype 2 variant isolates were tested for MICs with 21 antimicrobials. Resistance to ampicillin was the most common (57, 98.3%), followed by ticarcillin (56, 96.6%), tetracycline (55, 94.8%), chloramphenicol (54, 93.1%), trimethoprim/sulfamethoxazole (25, 43.1%), norfloxacin (22, 37.9%), cefazolin (22, 37.9%), ceftriaxone (20, 34.5%), cefoperazone (17, 29.3%), piperacillin (14, 24.1%), tobramycin (7, 12.1%), gentamicin (5, 8.6%), ticarcillin/clavulanic acid (3, 5.2%), cefoxitin (3, 5.2%), nitrofurantoin (2, 3.4%), aztreonam (2, 3.4%), and levofloxacin (1, 1.7%). None of the isolates were resistant to cefepime, imipenem, ceftazidime, and amikacin. In addition, strains intermediately resistant to ceftazidime, cefoperazone, levofloxacin, norfloxacin, piperacillin, tobramycin, gentamicin, aztreonam, and ticarcillin/clavulanic acid were also observed (Table 2). Furthermore, 57 of 58 *S. flexneri* serotype 2 variant isolates exhibited multidrug-resistant (MDR; resistant to three or more classes of antimicrobials), and only one strain from Shanghai was not. All of the isolates had 26 antibiotic-resistance profiles, dominated by resistance to TE/TIC/AMP/C (14/58, 24.1%), followed by TE/TIC/AMP/C/SXT (8/58, 13.8%), TE/TIC/AMP/C/SXT/NOR (5/58, 8.6%), and TE/TIC/AMP/C/NOR (3/58, 5.2%) (Table 3).

**Table 2:** **Comparison and antimicrobial susceptibility to 21 antibiotics among *S. flexneri* serotype 2 variant isolates from different regions**.

**Antibiotic**	**Antimicrobial resistance rate No. (%)**				
	**Total (*n* = 58)**	**Shanghai (*n* = 34)**	**Henan (*n* = 12)**	**Xinjiang (*n* = 8)**	**Guangxi (*n* = 4)**
Ceftazidime	0	0	0	0	0
Ceftriaxone	20 (34.5%)	12 (35.3%)	4 (33.3%)	4 (50%)	0
Piperacillin	14 (24.1%)	10 (29.4%)	2 (16.7%)	2 (25%)	0
Tetracycline	55 (94.8%)	31 (91.2%)	12 (100%)	8 (100%)	4 (100%)
Cefoperazone	17 (29.3%)	9 (26.5%)	4 (33.3%)	4 (50%)	0
Cefazolin	22 (37.9%)	14 (41.2%)	4 (33.3%)	4 (50%)	0
Ticarcillin	56 (96.6%)	33 (97.1%)	12 (100%)	7 (87.5%)	4 (100%)
Ticarcillin/clavulanic acid	3 (5.2%)	2 (5.9%)	0	1 (3.6%)	0
Aztreonam	2 (3.4%)	0	1 (8.3%)	1 (3.6%)	0
Ampicillin	57 (98.3%)	33 (97.1%)	12 (100%)	8 (100%)	4 (100%)
Chloramphenicol	54 (93.1%)	31 (91.2%)	12 (100%)	7 (87.5%)	4 (100%)
Trimethoprim/sulfamethoxazole	25 (43.1%)	16 (47.1%)	4 (33.3%)	3 (37.5%)	2 (50%)
Levofloxacin	1 (1.7%)	0	0	1 (12.5%)	0
Cefepime	0	0	0	0	0
Imipenem	0	0	0	0	0
nitrofurantoin	2 (3.4%)	2 (5.9%)	0	0	0
Cefoxitin	3 (5.2%)	3 (8.8%)	0	0	0
Tobramycin	7 (12.1%)	5 (14.7%)	2 (16.7%)	0	0
Gentamicin	5 (8.6%)	4 (11.2%)	1 (8.3%)	0	0
Norfloxacin	22 (37.9%)	16 (47.1%)	3 (25%)	3 (37.5%)	0
Amikacin	0	0	0	0	0

**Table 3:** **Dominant antimicrobial resistance profiles of 58 *Shigella flexneri* serotype 2 variant isolates from different regions of China**.

**Antimicrobial resistance profiles**	**Total No. (%) (*n* = 58)**	**Xinjiang No. (%) (*n* = 8)**	**Shanghai No. (%) (*n* = 34)**	**Henan No. (%) (*n* = 12)**	**Guangxi No. (%) (*n* = 4)**
TE/SXT	1 (1.7%)	0	1 (2.9%)	0	0
TE/TIC/AMP/C	14 (24.1%)	2 (25%)	6 (17.6%)	4 (33.3%)	2 (50%)
TIC/AMP/C/NOR	2 (3.4%)	0	2 (5.9%)	0	0
TE/TIC/AMP/C/SXT	8 (13.8%)	1 (12.5%)	2 (5.9%)	3 (25%)	2 (50%)
TE/TIC/AMP/C/NOR	3 (5.2%)	1 (12.5%)	2 (5.9%)	0	0
TIC/AMP/C/SXT/NOR	1 (1.7%)	0	1 (2.9%)	0	0
TE/GEN/TIC/AMP/C/SXT	1 (1.7%)	0	1 (2.9%)	0	0
TE/TIC/AMP/C/SXT/NOR	5 (8.6%)	0	5 (14.7%)	0	0
CRO/TE/CFZ/TIC/AMP/C	1 (1.7%)	0	1 (2.9%)	0	0
CRO/TE/CFP/CFZ/AMP/SXT	1 (1.7%)	1 (12.5%)	0	0	0
CRO/CFP/CFZ/TIC/TE/AMP/C	2 (3.4%)	0	0	2 (16.7%)	0
CRO/TE/CFZ/TIC/AMP/C/SXT	1 (1.7%)	0	1 (2.9%)	0	0
TE/TIC/AMP/C/TO/GEN/SXT/NOR	1 (1.7%)	0	0	1 (8.3%)	0
CRO/PIP/TE/CFP/CFZ/TIC/AMP/C	1 (1.7%)	0	1 (2.9%)	0	0
CRO/PIP/TE/CFP/CFZ/TIC/AMP/SXT	1 (1.7%)	0	1 (2.9%)	0	0
CRO/PIP/TE/CFP/CFZ/TIC/AMP/C/NOR	1 (1.7%)	0	1 (2.9%)	0	0
CRO/TE/CFP/CFZ/LEV/TIC/AMP/C/NOR	1 (1.7%)	1 (12.5%)	0	0	0
CRO/PIP/TE/CFP/CFZ//TOTIC/AMP/C/NOR	1 (1.7%)	0	0	1 (8.3%)	0
CRO/PIP/TE/CFZ/TO/TIC/TIM/AMP/C/NOR	1 (1.7%)	0	1 (2.9%)	0	0
CRO/PIP/TE/CFP/CFZ/TIC/ATM/AMP/C/SXT	1 (1.7%)	1 (12.5%)	0	0	0
CRO/PIP/TE/CFP/CFZ/TIC/TIM/AMP/C/NOR	2 (3.4%)	1 (12.5%)	0	1 (8.3%)	0
CRO/TE/CFZ/FOX/TO/TIC/AMP/C/SXT/NOR	2 (3.4%)	0	2 (5.9%)	0	0
CRO/PIP/TE/CFP/CFZ/FOX/TIC/AMP/C/NOR	1 (1.7%)	0	1 (2.9%)	0	0
CRO/TE/CFZ/FOX/TO/GEN/TIC/AMP/C/SXT/NOR	2 (3.4%)	0	2 (5.9%)	0	0
CRO/PIP/TE/CFP/CFZ/TO/GEN/TIC/TIM/AMP/C	1 (1.7%)	0	1 (2.9%)	0	0
CRO/PIP/TE/CFP/CFZ/TO/GEN/TIC/TIM/AMP/C/NOR	1 (1.7%)	0	1 (2.9%)	0	0
CRO/PIP/TE/CFP/CFZ/TO/GEN/TIC/AMP/C/SXT/NOR	1 (1.7%)	0	1 (2.9%)	0	0

Moreover, notable differences of antibiotic resistance profiles were also observed during the periods 2003–2008 and 2009–2013. Of the 58 serotype 2 variant strains, the resistance rate to cephalosporin and quinolones was 33.3% and 17.9% during 2003–2008, respectively, which increased to 47.4% and 78.9% during 2009–2013. The resistance rate to both cephalosporin and quinolones was 5.1% during 2003–2008, which increased to 42.1% during 2009–2013 (Table 4).

**Table 4:** **Resistance to quinolones or cephalosporin of 58 *S. flexneri* serotype 2 variant isolates from different regions of China during 2003–2008 and 2009–2013**.

**Resistance to quinolones or cephalosporin**	**No. (%) isolates**										***P*-value**
	**Total (*n* = 58)**		**Shanghai (*n* = 34)**		**Henan (*n* = 12)**		**Guangxi (*n* = 4)**		**Xinjiang (*n* = 8)**		
	**2003–2008 (*n* = 39)**	**2009–2013 (*n* = 19)**	**2003–2008 (*n* = 21)**	**2009–2013 (*n* = 13)**	**2003–2008 (*n* = 9)**	**2009–2013 (*n* = 3)**	**2003–2008 (*n* = 4)**	**2009–2013 (*n* = 0)**	**2003–2008 (*n* = 5)**	**2009–2013 (*n* = 3)**	
Non	21 (53.8)	3 (15.8)	9 (42.9)	1 (7.7)	6 (66.7)	1 (33.3)	4 (100)	0	2 (40)	1 (33.3)	≤0.01
Cephalosporin	13 (33.3)	9 (47.4)	8 (38.1)	6 (46.2)	3 (33.3)	1 (33.3)	0	0	2 (40)	2 (66.7)	0.3
Quinolones	7 (17.9)	15 (78.9)	4 (19)	12 (92.3)	3 (33.3)	2	0	0	2 (40)	1 (33.3)	≤0.01
Both	2 (5.1)	8 (42.1)	0	7 (53.8)	3 (33.3)	1 (33.3)	0	0	1 (20)	1 (33.3)	≤0.01

### MLST and PFGE analysis

**Figure 3:**
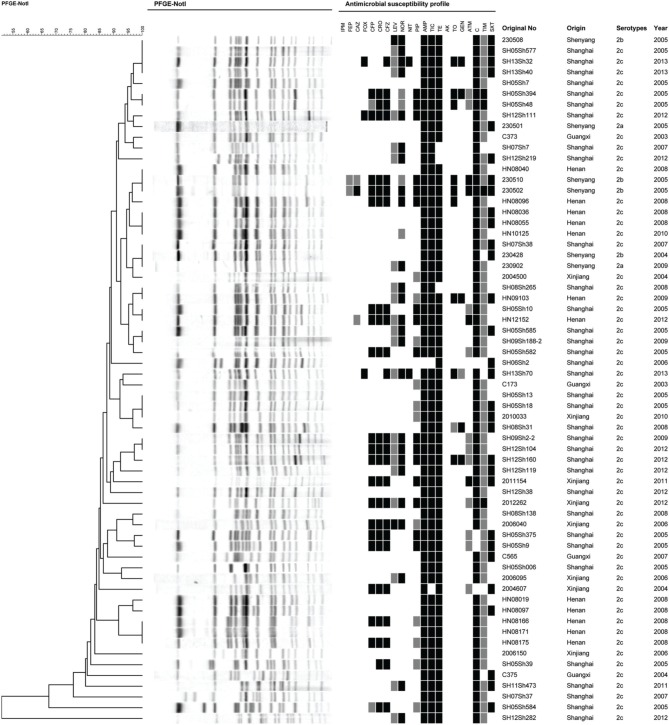
**Pulsed-field gel electrophoresis dendrogram and the antibiotic resistance profile of *S. flexneri* serotype 2 variant, 2a, and 2b isolates**. The original number, origin, serotype, and isolation year are indicated for each strain.

To determine the phylogenetic relationship of these serotype 2 variant strains, an extended MLST scheme of 15 genes was performed. We found that all the isolates in this study belonged to the same sequence type, which was designated ST100. ST100 also contained multiple other serotypes such as 1a, 1c, 2a, 2b, and Y, suggesting that ST100 has been circulating among different *S. flexneri* serotypes for a long time in China. PFGE was further performed to determine genetic relatedness among the isolates of serotype 2 variant and other serotypes. With a similarity of 80%, all the serotype 2 variant strains except two isolates (SH05Sh584 and SH12Sh282) formed a single cluster (containing 2a and 2b isolates). This result suggested that the serotype 2 variant strains were closely related to serotype 2a and 2b. Besides, the 58 *S. flexneri* serotype 2 variant isolates generated 45 PFGE patterns (Figure 3), suggesting a high genetic diversity among the serotype 2 variant strains.

### Molecular analysis of antibiotic-resistance determinants and integrons

Twenty-two of 58 isolates showing resistance to cephalosporin were selected to test for antibiotic-resistance determinants and integrons, including the *bla*_VIM_, *bla*_NDM_, *bla*_SHV_, *bla*_TEM_, *bla*_OXA_, *bla*_CTX−M_, *intI1*, and *intI2* gene regions (Table 5). PCR screenings showed that all strains were negative for *bla*_SHV_, *bla*_VIM_, and *bla*_NDM_, but positive for *bla*_TEM_, *bla*_OXA_, and *bla*_CTX−M_. Sequencing results using primers amplifying the whole *bla*_TEM_ gene showed 100% identity with the *bla*_TEM−1_ gene. All except two isolates from Shanghai and one isolate from Xinjiang harbored *bla*_OXA−1_. Besides, 18 isolates contained the *bla*_CTX−M_ gene; nine isolates from Shanghai, two from Xinjiang, and one from Henan harbored *bla*_CTX−M−14_; three isolates from Shanghai and one isolate from Henan harbored *bla*_CTX−M−3_; and one isolate from Xinjiang and one isolate from Henan harbored *bla*_CTX−M−79_. All isolates harbored class 1 integrons with *bla*_OXA−30_ and *aadA1* gene cassettes, and one isolate from Shanghai harbored *aacA4* and *cmlA1* gene cassettes. All of the isolates harbored class 2 integrons following *dfrA1*, *sat1*, and *aadA1* gene cassettes. In 22 quinolone-resistant isolates, no point mutations in QRDRs of *gyrB* and *parE* were found, but point mutations in QRDRs of *gyrA* and *parC* were identified in each resistant isolate. All of the norfloxacin-resistant isolates had the gyrA mutation of Ser83Leu and the parC mutations of Ser80Ile and His211Tyr in our study. Interestingly, the strain 2006040 (the only isolate resistant to levofloxacin) from Xinjiang and strain HN08096 from Henan had the gyrA mutation of Asp87Asn, while the rest of the strains except strain HN09103 had the gyrA mutation of Asp87Gly. Only two species of PMQR determinants [*qnrS* and *aac(6′)-Ib-cr*] were identified. All of the isolates harbored the *aac(6′)-Ib-cr* gene, and only two isolates from Shanghai harbored *qnrS*. Sequencing analysis determined that the two *qnrS* genes were *qnrS1* (Table 6).

**Table 5:** **Antibiogram and molecular analysis of the antibiotic-resistance determinants and integrons of *S. flexneri* serotype 2 variant isolates with resistance to cephalosporin**.

**Strain No**.	**Antibiogram**	**Antibiotic-resistant determinants and integrons present**
**STRAINS RESISTANT TO CEPHALOSPORIN**		
2004607	CRO/TE/CFP/CFZ/AMP/SXT	*IntI1* (*bla*_OXA-30_+*aadA1*)*, IntI2* (*dfrA1+sat1+aadA1*), *bla*_TEM1_
SH05Sh584	CRO/TE/CFZ/TIC/AMP/C/SXT	*IntI1* (*bla*_OXA-30_+*aadA1*)*, IntI2* (*dfrA1+sat1+aadA1*), *bla*_OXA1_, *bla*_TEM1_, *bla*_CTX-M-14_
HN08166	CRO/TE/CFP/CFZ/TIC/AMP/C	*IntI1* (*bla*_OXA-30_+*aadA1*)*, IntI2* (*dfrA1+sat1+aadA1*), *bla*_OXA1_, *bla*_TEM1_, *bla*_CTX-M-14_
HN08175	CRO/TE/CFP/CFZ/TIC/AMP/C	*IntI1* (*bla*_OXA-30_+*aadA1*)*, IntI2* (*dfrA1+sat1+aadA1*), *bla*_OXA1_, *bla*_TEM1_
SH05Sh582	CRO/PIP/TE/CFP/CFZ/TIC/AMP/C	*IntI1* (*bla*_OXA-30_+*aadA1*)*, IntI2* (*dfrA1+sat1+aadA1*), *bla*_OXA1_, *bla*_TEM1_, *bla*_CTX-M-3_
SH05Sh10	CRO/PIP/TE/CFP/CFZ/TIC/AMP/C	*IntI1* (*bla*_OXA-30_+*aadA1*)*, IntI2* (*dfrA1+sat1+aadA1*), *bla*_OXA1_, *bla*_TEM1_, *bla*_CTX-M-14_
SH05Sh9	CRO/PIP/TE/CFP/CFZ/TIC/AMP/SXT	*IntI1* (*bla*_OXA-30_+*aadA1*)*, IntI2* (*dfrA1+sat1+aadA1*), *bla*_TEM1_, *bla*_CTX-M-14_
SH05sh375	CRO/PIP/TE/CFP/CFZ/TIC/AMP/SXT	*IntI1* (*bla*_OXA-30_+*aadA1*)*, IntI2* (*dfrA1+sat1+aadA1*), *bla*_TEM1_, *bla*_CTX-M-14_
SH05Sh39	CRO/PIP/TE/CFP/CFZ/TIC/AMP/SXT	*IntI1* (*bla*_OXA-30_+*aadA1*)*, IntI2* (*dfrA1+sat1+aadA1*), *bla*_OXA1_, *bla*_TEM1_, *bla*_CTX-M-14_
SH09sh2-2	CRO/PIP/TE/CFP/CFZ/TIC/AMP/C/NOR	*IntI1* (*bla*_OXA-30_+*aadA1*)*, IntI2* (*dfrA1+sat1+aadA1*), *bla*_OXA1_, *bla*_TEM1_, *bla*_CTX-M-14_
SH12Sh104	CRO/PIP/TE/CFP/CFZ/TIC/AMP/C/NOR	*IntI1* (*bla*_OXA-30_+*aadA1*)*, IntI2* (*dfrA1+sat1+aadA1*), *bla*_OXA1_, *bla*_TEM1_, *bla*_CTX-M-14_
2006040	CRO/TE/CFP/CFZ/LEV/TIC/AMP/C/NOR	*IntI1* (*bla*_OXA-30_+*aadA1*)*, IntI2* (*dfrA1+sat1+aadA1*), *bla*_OXA1_, *bla*_TEM1_, *bla*_CTX-M-14_
SH05sh48	CRO/PIP/TE/CFZ/TO/TIC/TIM/AMP/C	*IntI1* (*bla*_OXA-30_+*aadA1*)*, IntI2* (*dfrA1+sat1+aadA1*), *bla*_OXA1_, *bla*_TEM1_, *bla*_CTX-M-3_
2012262	CRO/PIP/TE/CFP/CFZ/TIC/TIM/AMP/C/NOR	*IntI1* (*bla*_OXA-30_+*aadA1*)*, IntI2* (*dfrA1+sat1+aadA1*), *bla*_OXA1_, *bla*_TEM1_, *bla*_CTX-M-14_
HN08096	CRO/PIP/TE/CFP/CFZ/TO/TIC/AMP/C/NOR	*IntI1* (*bla*_OXA-30_+*aadA1*)*, IntI2* (*dfrA1+sat1+aadA1*), *bla*_OXA1_, *bla*_TEM1_, *bla*_CTX-M-3_
SH13Sh70	CRO/TE/CFZ/FOX/TO/TIC/AMP/C/SXT/NOR	*IntI1* (*bla*_OXA-30_+*aadA1*)*, IntI2* (*dfrA1+sat1+aadA1*), *bla*_OXA1_, *bla*_TEM1_
SH12Sh111	CRO/PIP/TE/CFP/CFZ/FOX/TIC/AMP/C/NOR	*IntI1* (*bla*_OXA-30_+*aadA1*)*, IntI2* (*dfrA1+sat1+aadA1*), *bla*_OXA1_, *bla*_TEM1_, *bla*_CTX-M-14_
2011154	CRO/PIP/TE/CFP/CFZ/TIC/ATM/AMP/C/SXT	*IntI1* (*bla*_OXA-30_+*aadA1*)*, IntI2* (*dfrA1+sat1+aadA1*), *bla*_OXA1_, *bla*_TEM1_, *bla*_CTX-M-79_
HN12152	CRO/PIP/TE/CFP/CFZ/TIC/ATM/AMP/C/NOR	*IntI1* (*bla*_OXA-30_+*aadA1*)*, IntI2* (*dfrA1+sat1+aadA1*), *bla*_OXA1_, *bla*_TEM1_, *bla*_CTX-M-79_
SH05sh394	CRO/PIP/TE/CFP/CFZ/TO/GEN/TIC/TIM/AMP/C	*IntI1* (*bla*_OXA-30_+*aadA1*)*, IntI2* (*dfrA1+sat1+aadA1*), *bla*_OXA1_, *bla*_TEM1_, *bla*_CTX-M-3_
SH13Sh32	NIT/TE/CFZ/FOX/TO/GEN/TIC/AMP/C/SXT/NOR	*IntI1* (*bla*_OXA-30_+*aadA1*)*, IntI2* (*dfrA1+sat1+aadA1*), *bla*_OXA1_, *bla*_TEM1_
SH12Sh160	CRO/PIP/TE/CFP/CFZ/TO/GEN/TIC/AMP/C/SXT/NOR	*IntI1* (*bla*_OXA-30_+*aadA1*; *aacA4+cmlA1*)*, IntI2* (*dfrA1+sat1+aadA1*), *bla*_OXA1_, *bla*_TEM1_, *bla*_CTX-M-14_

**Table 6:** **Antibiogram and molecular analysis of the antibiotic-resistance determinants and integrons of *S. flexneri* serotype 2 variant isolates with resistance to fluoroquinolone**.

**Strain No**.	**Antibiogram**	**PMQR determinant**		**Amino acid in QRDR**			
				***parC***		***gyrA***	
		***aac(6′)-Ib-cr***	***qnrS***	***80Ser***	**83Ser**	**87Asp**	**211His**
**STRAINS RESISTANT TO FLUOROQUINOLONE**							
SH07Sh007	TIC/AMP/C/NOR	+	–	Ile	Leu	Gly	Tyr
SH08Sh265	TIC/AMP/C/NOR	+	–	Ile	Leu	Gly	Tyr
2006095	TE/TIC/AMP/C/NOR	+	–	Ile	Leu	Gly	Tyr
SH12Sh282	TE/TIC/AMP/C/NOR	+	–	Ile	Leu	Gly	Tyr
SH09sh188-2	TE/TIC/AMP/C/NOR	+	–	Ile	Leu	Gly	Tyr
SH12Sh219	TIC/AMP/C/SXT/NOR	+	–	Ile	Leu	Gly	Tyr
SH05sh577	TE/TIC/AMP/C/SXT/NOR	+	+	Ile	Leu	Gly	Tyr
SH11sh473	TE/TIC/AMP/C/SXT/NOR	+	–	Ile	Leu	Gly	Tyr
SH12Sh119	TE/TIC/AMP/C/SXT/NOR	+	–	Ile	Leu	Gly	Tyr
SH13Sh40	TE/TIC/AMP/C/SXT/NOR	+	–	Ile	Leu	Gly	Tyr
SH05sh585	TE/TIC/AMP/C/SXT/NOR	+	–	Ile	Leu	Gly	Tyr
HN09103	TE/TO/GEN/TIC/AMP/C/SXT/NOR	+	–	Ile	Leu	Asp	Tyr
SH09sh2-2	CRO/PIP/TE/CFP/CFZ/TIC/AMP/C/NOR	+	–	Ile	Leu	Gly	Tyr
SH12Sh104	CRO/PIP/TE/CFP/CFZ/TIC/AMP/C/NOR	+	–	Ile	Leu	Gly	Tyr
2006040	CRO/TE/CFP/CFZ/LEV/TIC/AMP/C/NOR	+	–	Ile	Leu	Asn	Tyr
SH13Sh70	CRO/TE/CFZ/FOX/TO/TIC/AMP/C/SXT/NOR	+	–	Ile	Leu	Gly	Tyr
HN08096	CRO/PIP/TE/CFP/CFZ/TO/TIC/AMP/C/NOR	+	–	Ile	Leu	Asn	Tyr
HN12152	CRO/PIP/TE/CFP/CFZ/TIC/ATM/AMP/C/NOR	+	–	Ile	Leu	Gly	Tyr
2012262	CRO/PIP/TE/CFP/CFZ/TIC/TIM/AMP/C/NOR	+	–	Ile	Leu	Gly	Tyr
SH12Sh111	CRO/PIP/TE/CFP/CFZ/FOX/TIC/AMP/C/NOR	+	+	Ile	Leu	Gly	Tyr
SH13Sh32	NIT/TE/CFZ/FOX/TO/GEN/TIC/AMP/C/SXT/NOR	+	–	Ile	Leu	Gly	Tyr
SH12Sh160	CRO/PIP/TE/CFP/CFZ/TO/GEN/TIC/AMP/C/SXT/NOR	+	–	Ile	Leu	Gly	Tyr

## Discussion

The emergence of MDR strains has become a serious problem and has complicated the selection of empirical treatment for shigellosis. In the present study, the serotype 2 variant isolates were highly resistant to ampicillin, chloramphenicol, tetracycline, and ticarcillin, and 98.3% of these isolates showed MDR profiles. One of the reasons for the rapid accumulation of resistance is excessive or inappropriate use of antibiotics in outpatients in China (Pickering, 2004; Zhang et al., 2011).

Our study further indicates that current resistance patterns have changed and that empirical therapy should keep pace with these changes. According to previous results, fluoroquinolones and third-generation cephalosporins are probably the best choice for empiric treatment of severe gastrointestinal infections caused by pathogenic bacteria (Gendrel et al., 2008). However, the resistance rates of these isolates to fluoroquinolones and third-generation cephalosporins in the serotype 2 variant strains have increased at an alarming rate, and continuous surveillance of the resistance pattern will be essential to choose the appropriate antimicrobial therapy.

The increasing antibiotic resistance rate led us to study the genetics and mechanisms of antibiotic resistance. The horizontal transfer of integrons may account for the dissemination of resistance genes, and resistance to some antibiotics is associated with the presence of class 1 and class 2 integrons containing resistance gene cassettes (Ke et al., 2011). In the current study, *bla*_OXA−30_ and *aadA1* were detected in the gene cassettes of class 1 integrons, and *dfrA1+sat1+aadA1* was detected in the gene cassettes of class 2 integrons. In addition, of the 22 cephalosporin-resistant isolates, 100% harbored the *bla*_TEM−1_ resistance gene, 81.8% harbored the *bla*_CTX_ resistance gene, and 90.9% harbored the *bla*_OXA_ resistance gene. Additionally, the strong ability to acquire resistance genes facilitated the survival of bacteria under the pressure of various antibiotics. The *bla*_TEM−1_ gene was detected in all of the ESBL-producing isolates; this gene exists at high frequencies in antibiotic-resistant bacteria across the globe and confers resistance to penicillin and other β-lactamase antibiotics (Barlow and Hall, 2003). OXA-type β-lactamases confer resistance to ampicillin and cephalothin, and they are characterized by high hydrolytic activity against oxacillin and cloxacillin (Bradford, 2001). Sequencing analysis showed that all of the *bla*_OXA_ genes were *bla*_OXA−1_, indicating the host specificity for this subtype in *S. flexneri*, which is consistent with the result of a previous study (Siu et al., 2000).

Fluoroquinolone resistance in *Enterobacteriaceae* has been mostly attributed to modifications in the QRDRs of the *gyr*A and *par*C genes and/or PMQR determinants such as *qnr* and [*aac(6′)-Ib-cr*] (Folster et al., 2011). In the current study, 95% of the quinolone-resistant *Shigella* isolates had at least three point mutations in *gyr*A (Ser83Leu, Asp87Gly/Asn, and His211Tyr) and *par*C (Ser80Ile). Furthermore, different amino acid substitutions at the same position may result in different quinolone susceptibility levels (Ruiz, 2003). The His211Tyr mutation in gyrA was detected in resistant *S. flexneri* serotype 2a strains from Bangladesh (Azmi et al., 2014). Notably, all of the quinolone-resistant isolates in our study had the gyrA mutation of His211Tyr. PMQR determinants are located on mobile genetic elements (i.e., plasmids), which may allow for dissemination among *Shigella* (Folster et al., 2011). The presence of the *qnr* gene can facilitate the selection of chromosomal mutations that cause quinolone resistance (Jacoby et al., 2003; Martinez-Martinez et al., 2003). Worldwide, *S. flexneri* serotypes 1a, 2a, 2b, and 4c carrying the *qnrS* gene were reported with low incidence (Hata et al., 2005; Pu et al., 2009). In our study, only two strains from Shanghai carried the *qnrS1* gene with a percentage of 3.4%, and one of the *qnrS*-positive strains was the only isolate that was resistant to levofloxacin. The *aac(6′)-Ib-cr* gene is identified in many Enterobacteriaceae and is responsible for low-level resistance to fluoroquinolones (Frasson et al., 2011). The *aac(6′)-Ib-cr*–positive *S. flexneri* 2a strain was first isolated in 1998 (Pu et al., 2009), and all of the 17 quinolone-resistant isolates were *aac(6′)-Ib-cr*–positive, suggesting that the *aac(6′)-Ib-cr* gene had been present in China for many years.

Serotype conversion is a major mechanism for *S. flexneri* to escape protective host immune responses, which may drive the emergence of novel and atypical serotypes under pressure (Allison and Verma, 2000). Based on phenotypic and genotypic analysis, the serotype 2 variant is likely to originate from serotypes 2a and 2b, which have experienced changes in their genome to adapt to altered environmental conditions. The serotype 2 variant was circulated as a major serotype during our 11-year study period, and the widespread dissemination of this serotype poses a serious threat to public health not only in China but also in the world.

In summary, studies on the serotype 2 variant have become very important owing to the increased epidemic frequency and the prevalence of MDR strains. In view of the fact that this serotype was prevalent in several provinces in China, it is likely to provoke a major crisis because MDR clones can and may have already spread to other countries. Therefore, continuous surveillance will be needed to determine the distribution and resistance development of this serotype so as to understand the actual disease burden and provide guidance for the clinical treatment of shigellosis.

### Conflict of interest statement

The authors declare that the research was conducted in the absence of any commercial or financial relationships that could be construed as a potential conflict of interest.

## References

[B1] Ahmed A. M. Furuta K. Shimomura K. Kasama Y. Shimamoto T. (2006). Genetic characterization of multidrug resistance in Shigella spp. from Japan. J. Med. Microbiol. 55, 1685–1691. 10.1099/jmm.0.46725-0 17108272

[B2] Allison G. E. Verma N. K. (2000). Serotype-converting bacteriophages and O-antigen modification in *Shigella flexneri*. Trends Microbiol. 8, 17–23. 10.1016/S0966-842X(99)01646-7 10637639

[B3] Azmi I. J. Khajanchi B. K. Akter F. Hasan T. N. Shahnaij M. Akter M.. (2014). Fluoroquinolone resistance mechanisms of *Shigella flexneri* isolated in Bangladesh. PLoS ONE 9:e102533. 10.1371/journal.pone.0102533 25028972 PMC4100904

[B4] Barlow M. Hall B. G. (2003). Experimental prediction of the natural evolution of antibiotic resistance. Genetics 163, 1237–1241. 12702671 10.1093/genetics/163.4.1237 PMC1462515

[B5] Bradford P. A. (2001). Extended-spectrum beta-lactamases in the 21st century: characterization, epidemiology, and detection of this important resistance threat. Clin. Microbiol. Rev. 14, 933–951. 10.1128/CMR.14.4.933-951.2001 11585791 PMC89009

[B6] Carlin N. I. Rahman M. Sack D. A. Zaman A. Kay B. Lindberg A. A. (1989). Use of monoclonal antibodies to type *Shigella flexneri* in Bangladesh. J. Clin. Microbiol. 27, 1163–1166. 2666435 10.1128/jcm.27.6.1163-1166.1989 PMC267520

[B7] El-Gendy A. El-Ghorab N. Lane E. M. Elyazeed R. A. Carlin N. I. Mitry M. M.. (1999). Identification of *Shigella flexneri* subserotype 1c in rural Egypt. J. Clin. Microbiol. 37, 873–874. 9986881 10.1128/jcm.37.3.873-874.1999 PMC84593

[B8] Folster J. P. Pecic G. Bowen A. Rickert R. Carattoli A. Whichard J. M. (2011). Decreased susceptibility to ciprofloxacin among Shigella isolates in the United States, 2006 to 2009. Antimicrob. Agents Chemother. 55, 1758–1760. 10.1128/AAC.01463-10 21220535 PMC3067149

[B9] Frasson I. Cavallaro A. Bergo C. Richter S. N. Palu G. (2011). Prevalence of aac(6′)-Ib-cr plasmid-mediated and chromosome-encoded fluoroquinolone resistance in Enterobacteriaceae in Italy. Gut Pathog. 3:12. 10.1186/1757-4749-3-12 21827661 PMC3163596

[B10] Galani I. Souli M. Mitchell N. Chryssouli Z. Giamarellou H. (2010). Presence of plasmid-mediated quinolone resistance in *Klebsiella pneumoniae* and *Escherichia coli* isolates possessing blaVIM-1 in Greece. Int. J. Antimicrob. Agents 36, 252–254. 10.1016/j.ijantimicag.2010.05.004 20580536

[B11] Gaynor K. Park S. Y. Kanenaka R. Colindres R. Mintz E. Ram P. K.. (2009). International foodborne outbreak of *Shigella sonnei* infection in airline passengers. Epidemiol. Infect. 137, 335–341. 10.1017/S0950268807000064 18177516

[B12] Gendrel D. Cohen R. European Society for Pediatric Infectious, D., European Society for Gastroenterology, H., and Nutrition (2008). [Bacterial diarrheas and antibiotics: european recommendations]. Arch. Pediatr. 15(Suppl 2), S93–S96. 10.1016/S0929-693X(08)74223-4 19000862

[B13] Hata M. Suzuki M. Matsumoto M. Takahashi M. Sato K. Ibe S.. (2005). Cloning of a novel gene for quinolone resistance from a transferable plasmid in *Shigella flexneri* 2b. Antimicrob. Agents Chemother. 49, 801–803. 10.1128/AAC.49.2.801-803.2005 15673773 PMC547361

[B14] Hu L. F. Li J. B. Ye Y. Li X. (2007). Mutations in the GyrA subunit of DNA gyrase and the ParC subunit of topoisomerase IV in clinical strains of fluoroquinolone-resistant Shigella in Anhui, China. J. Microbiol. 45, 168–170. 17483803

[B15] Huang I. F. Chiu C. H. Wang M. H. Wu C. Y. Hsieh K. S. Chiou C. C. (2005). Outbreak of dysentery associated with ceftriaxone-resistant *Shigella sonnei*: first report of plasmid-mediated CMY-2-type AmpC beta-lactamase resistance in *S. sonnei.* J. Clin. Microbiol. 43, 2608–2612. 10.1128/JCM.43.6.2608-2612.2005 15956372 PMC1151904

[B16] Jacoby G. A. Chow N. Waites K. B. (2003). Prevalence of plasmid-mediated quinolone resistance. Antimicrob. Agents Chemother. 47, 559–562. 10.1128/AAC.47.2.559-562.2003 12543659 PMC151764

[B17] Ke X. Gu B. Pan S. Tong M. (2011). Epidemiology and molecular mechanism of integron-mediated antibiotic resistance in Shigella. Arch. Microbiol. 193, 767–774. 10.1007/s00203-011-0744-3 21842348

[B18] Kotloff K. L. Winickoff J. P. Ivanoff B. Clemens J. D. Swerdlow D. L. Sansonetti P. J.. (1999). Global burden of Shigella infections: implications for vaccine development and implementation of control strategies. Bull. World Health Organ. 77, 651–666. 10516787 PMC2557719

[B19] Kuo H. W. Kasper S. Jelovcan S. Hoger G. Lederer I. Konig C.. (2009). A food-borne outbreak of *Shigella sonnei* gastroenteritis, Austria, 2008. Wien. Klin. Wochenschr. 121, 157–163. 10.1007/s00508-008-1141-7 19280143

[B20] Martinez-Martinez L. Pascual A. Garcia I. Tran J. Jacoby G. A. (2003). Interaction of plasmid and host quinolone resistance. J. Antimicrob. Chemother. 51, 1037–1039. 10.1093/jac/dkg157 12654766

[B21] Matar G. M. Jaafar R. Sabra A. Hart C. A. Corkill J. E. Dbaibo G. S.. (2007). First detection and sequence analysis of the bla-CTX-M-15 gene in Lebanese isolates of extended-spectrum-beta-lactamase-producing *Shigella sonnei*. Ann. Trop. Med. Parasitol. 101, 511–517. 10.1179/136485907X193860 17716434

[B22] Mathers C. D. Boerma T. Ma Fat D. (2009). Global and regional causes of death. Br. Med. Bull. 92, 7–32. 10.1093/bmb/ldp028 19776034

[B23] Pan J.-C. Ye R. Meng D.-M. Zhang W. Wang H.-Q. Liu K.-Z. (2006). Molecular characteristics of class 1 and class 2 integrons and their relationships to antibiotic resistance in clinical isolates of *Shigella sonnei* and *Shigella flexneri*. J. Antimicrob. Chemother. 58, 288–296. 10.1093/jac/dkl228 16766536

[B24] Pickering L. K. (2004). Antimicrobial resistance among enteric pathogens. Semin. Pediatr. Infect. Dis. 15, 71–77. 10.1053/j.spid.2004.01.009 15185189

[B25] Pu X. Y. Pan J. C. Wang H. Q. Zhang W. Huang Z. C. Gu Y. M. (2009). Characterization of fluoroquinolone-resistant *Shigella flexneri* in Hangzhou area of China. J. Antimicrob. Chemother. 63, 917–920. 10.1093/jac/dkp087 19297378

[B26] Qiu S. Wang Y. Xu X. Li P. Hao R. Yang C.. (2013). Multidrug-resistant atypical variants of *Shigella flexneri* in China. Emerging Infect. Dis. 19, 1147–1150. 10.3201/eid1907.121221 23763754 PMC3713959

[B27] Qiu S. Wang Z. Chen C. Liu N. Jia L. Liu W.. (2011). Emergence of a novel *Shigella flexneri* serotype 4s strain that evolved from a serotype X variant in China. J. Clin. Microbiol. 49, 1148–1150. 10.1128/JCM.01946-10 21177890 PMC3067715

[B28] Qu F. Bao C. Chen S. Cui E. Guo T. Wang H.. (2012). Genotypes and antimicrobial profiles of *Shigella sonnei* isolates from diarrheal patients circulating in Beijing between 2002 and 2007. Diagn. Microbiol. Infect. Dis. 74, 166–170. 10.1016/j.diagmicrobio.2012.06.026 22858547 PMC7127854

[B29] Robicsek A. Strahilevitz J. Sahm D. F. Jacoby G. A. Hooper D. C. (2006). qnr prevalence in ceftazidime-resistant Enterobacteriaceae isolates from the United States. Antimicrob. Agents Chemother. 50, 2872–2874. 10.1128/AAC.01647-05 16870791 PMC1538681

[B30] Ruiz J. (2003). Mechanisms of resistance to quinolones: target alterations, decreased accumulation and DNA gyrase protection. J. Antimicrob. Chemother. 51, 1109–1117. 10.1093/jac/dkg222 12697644

[B31] Siu L. K. Lo J. Y. Yuen K. Y. Chau P. Y. Ng M. H. Ho P. L. (2000). beta-lactamases in *Shigella flexneri* isolates from Hong Kong and Shanghai and a novel OXA-1-like beta-lactamase, OXA-30. Antimicrob. Agents Chemother. 44, 2034–2038. 10.1128/AAC.44.8.2034-2038.2000 10898672 PMC90010

[B32] Stagg R. M. Cam P. D. Verma N. K. (2008). Identification of newly recognized serotype 1c as the most prevalent *Shigella flexneri* serotype in northern rural Vietnam. Epidemiol. Infect. 136, 1134–1140. 10.1017/S0950268807009600 17922932 PMC2870902

[B33] Sui J. Zhang J. Sun J. Chang Z. Zhang W. Wang Z. (2010). Surveillance of bacillary dysentery in China, 2009. Dis. Surveill. 25, 947–950 10.3784/j.issn.1003-9961.2010.12.006

[B34] Sun Q. Lan R. Wang J. Xia S. Wang Y. Wang Y.. (2013). Identification and characterization of a novel *Shigella flexneri* serotype Yv in China. PLoS ONE 8:e70238. 10.1371/journal.pone.0070238 23936172 PMC3728103

[B35] Talukder K. A. Dutta D. K. Safa A. Ansaruzzaman M. Hassan F. Alam K.. (2001). Altering trends in the dominance of *Shigella flexneri* serotypes and emergence of serologically atypical *S. flexneri* strains in Dhaka, Bangladesh. J. Clin. Microbiol. 39, 3757–3759. 10.1128/JCM.39.10.3757-3759.2001 11574611 PMC88427

[B36] Tariq A. Haque A. Ali A. Bashir S. Habeeb M. A. Salman M.. (2012). Molecular profiling of antimicrobial resistance and integron association of multidrug-resistant clinical isolates of Shigella species from Faisalabad, Pakistan. Can. J. Microbiol. 58, 1047–1054. 10.1139/w2012-085 22906205

[B37] Ye C. Lan R. Xia S. Zhang J. Sun Q. Zhang S.. (2010). Emergence of a new multidrug-resistant serotype X variant in an epidemic clone of *Shigella flexneri*. J. Clin. Microbiol. 48, 419–426. 10.1128/JCM.00614-09 19955273 PMC2815595

[B38] Zhang W. Luo Y. Li J. Lin L. Ma Y. Hu C.. (2011). Wide dissemination of multidrug-resistant Shigella isolates in China. J. Antimicrob. Chemother. 66, 2527–2535. 10.1093/jac/dkr341 21859815

