# Supplementary material for: Prevalence and antimicrobial resistance of Shigella flexneri serotype 2 variant in China
Source: Front Microbiol. 2015 May 7;6:435. doi: 10.3389/fmicb.2015.00435 (PMC4423435; doi:10.3389/fmicb.2015.00435)
Supplement: Supplementary file 1 [file Table1.DOCX]

**Supplementary Table 1.** The information of *Shigella flexneri* serotype 2 variant isolates isolated from diarrheal patients

| **Original No.** | **Species** | **Serotype** | **Source** | **Origin** | **Isolation time** |
| --- | --- | --- | --- | --- | --- |
| SH05Sh10 | *S. flexneri* | 2 variant | Human | Shanghai | 2005 |
| SH05Sh13 | *S. flexneri* | 2 variant | Human | Shanghai | 2005 |
| SH05Sh18 | *S. flexneri* | 2 variant | Human | Shanghai | 2005 |
| SH05Sh375 | *S. flexneri* | 2 variant | Human | Shanghai | 2005 |
| SH05Sh39 | *S. flexneri* | 2 variant | Human | Shanghai | 2005 |
| SH05Sh394 | *S. flexneri* | 2 variant | Human | Shanghai | 2005 |
| SH05Sh48 | *S. flexneri* | 2 variant | Human | Shanghai | 2005 |
| SH05Sh577 | *S. flexneri* | 2 variant | Human | Shanghai | 2005 |
| SH05Sh582 | *S. flexneri* | 2 variant | Human | Shanghai | 2005 |
| SH05Sh584 | *S. flexneri* | 2 variant | Human | Shanghai | 2005 |
| SH05Sh585 | *S. flexneri* | 2 variant | Human | Shanghai | 2005 |
| SH05Sh7 | *S. flexneri* | 2 variant | Human | Shanghai | 2005 |
| SH05Sh9 | *S. flexneri* | 2 variant | Human | Shanghai | 2006 |
| 2006150 | *S. flexneri* | 2 variant | Human | Xinjiang | 2006 |
| SH06Sh2 | *S. flexneri* | 2 variant | Human | Shanghai | 2007 |
| SH07Sh37 | *S. flexneri* | 2 variant | Human | Shanghai | 2007 |
| SH07Sh38 | *S. flexneri* | 2 variant | Human | Shanghai | 2007 |
| SH08Sh138 | *S. flexneri* | 2 variant | Human | Shanghai | 2008 |
| SH08Sh265 | *S. flexneri* | 2 variant | Human | Shanghai | 2008 |
| SH08Sh31 | *S. flexneri* | 2 variant | Human | Shanghai | 2008 |
| SH09Sh188-2 | *S. flexneri* | 2 variant | Human | Shanghai | 2009 |
| SH09Sh2-2 | *S. flexneri* | 2 variant | Human | Shanghai | 2009 |
| SH11Sh473 | *S. flexneri* | 2 variant | Human | Shanghai | 2011 |
| SH12Sh104 | *S. flexneri* | 2 variant | Human | Shanghai | 2012 |
| SH12Sh111 | *S. flexneri* | 2 variant | Human | Shanghai | 2012 |
| SH12Sh119 | *S. flexneri* | 2 variant | Human | Shanghai | 2012 |
| SH12Sh160 | *S. flexneri* | 2 variant | Human | Shanghai | 2012 |
| SH12Sh219 | *S. flexneri* | 2 variant | Human | Shanghai | 2012 |
| SH12Sh282 | *S. flexneri* | 2 variant | Human | Shanghai | 2012 |
| SH12Sh38 | *S. flexneri* | 2 variant | Human | Shanghai | 2012 |
| SH13Sh32 | *S. flexneri* | 2 variant | Human | Shanghai | 2013 |
| SH13Sh40 | *S. flexneri* | 2 variant | Human | Shanghai | 2013 |
| SH13Sh70 | *S. flexneri* | 2 variant | Human | Shanghai | 2013 |
| XJ2004500 | *S. flexneri* | 2 variant | Human | Xinjiang | 2004 |
| XJ2004607 | *S. flexneri* | 2 variant | Human | Xinjiang | 2004 |
| XJ2006040 | *S. flexneri* | 2 variant | Human | Xinjiang | 2006 |
| XJ2010033 | *S. flexneri* | 2 variant | Human | Xinjiang | 2010 |
| XJ2011154 | *S. flexneri* | 2 variant | Human | Xinjiang | 2011 |
| XJ2012262 | *S. flexneri* | 2 variant | Human | Xinjiang | 2012 |
| XJ2006095 | *S. flexneri* | 2 variant | Human | Xinjiang | 2006 |
| C173 | *S. flexneri* | 2 variant | Human | Guangxi | 2003 |
| C373 | *S. flexneri* | 2 variant | Human | Guangxi | 2004 |
| C375 | *S. flexneri* | 2 variant | Human | Guangxi | 2004 |
| C565 | *S. flexneri* | 2 variant | Human | Guangxi | 2007 |
| SH07Sh7 | *S. flexneri* | 2 variant | Human | Shanghai | 2007 |
| SH05Sh6 | *S. flexneri* | 2 variant | Human | Shanghai | 2005 |
| HN08019 | *S. flexneri* | 2 variant | Human | Henan | 2008 |
| HN08036 | *S. flexneri* | 2 variant | Human | Henan | 2008 |
| HN08040 | *S. flexneri* | 2 variant | Human | Henan | 2008 |
| HN08055 | *S. flexneri* | 2 variant | Human | Henan | 2008 |
| HN08096 | *S. flexneri* | 2 variant | Human | Henan | 2008 |
| HN08097 | *S. flexneri* | 2 variant | Human | Henan | 2008 |
| HN08166 | *S. flexneri* | 2 variant | Human | Henan | 2008 |
| HN08171 | *S. flexneri* | 2 variant | Human | Henan | 2008 |
| HN08175 | *S. flexneri* | 2 variant | Human | Henan | 2008 |
| HN09103 | *S. flexneri* | 2 variant | Human | Henan | 2009 |
| HN10125 | *S. flexneri* | 2 variant | Human | Henan | 2010 |
| HN12152 | *S. flexneri* | 2 variant | Human | Henan | 2012 |
